# Configurational Paths to Higher Efficiency in County Hospital: Evidence From Qualitative Comparative Analysis

**DOI:** 10.3389/fpubh.2022.918571

**Published:** 2022-06-10

**Authors:** Gang Yin, Jie Ning, Yarui Peng, Jingkai Yue, Hongbing Tao

**Affiliations:** ^1^Department of Health Administration, School of Medicine and Health Management, Tongji Medical College, Huazhong University of Science and Technology, Wuhan, China; ^2^Sun Yat-sen University Cancer Center, Guangzhou, China; ^3^State Key Laboratory of Oncology in South China, Guangzhou, China; ^4^Collaborative Innovation Center for Cancer Medicine, Guangzhou, China; ^5^The Central Hospital of Wuhan, Tongji Medical College, Huazhong University of Science and Technology, Wuhan, China

**Keywords:** county hospital, hospital efficiency, configurational paths, qualitative comparative analysis, QCA

## Abstract

**Background:**

The efficient operation of county-level medical institutions is a significant guarantee in constructing Chinese rural tertiary care service networks. However, it is still unclear how to increase the efficiency of county hospitals under the interaction of multiple factors. In this study, 35 county general hospitals in China were selected to explore the configuration paths of county hospitals' high and poor efficiency status under the Environment-Structure-Behavior (ESB) framework and provide evidence-based recommendations for measures to enhance its efficiency.

**Methods:**

Data envelopment analysis with the bootstrapping procedure was used to estimate the technical efficiency value of case hospitals. A fuzzy-set qualitative comparative analysis approach was carried out to explore the configuration of conditions to the efficiency status.

**Results:**

Antecedent configurations affecting the efficiency status of county hospitals were identified based on the ESB analytical framework. Three high-efficiency configuration paths can be summarized as structural optimization, capacity enhancement, and government support. Another three types of paths, namely insufficient capacity, aggressive expansion, and poor decision-making, will lead to inefficient configurations.

**Conclusion:**

Qualitative comparative analysis is necessary when exploring complex causality. The efficiency situation of county hospitals results from a combination of influencing factors instead of the effect of a single one. There is no solitary configuration for high efficiency that applies to all healthcare units. Any measures aimed at efficiency promotion should be discussed within the framework of a case-specific analysis.

## Introduction

The three-tier medical and health service system in rural China, consisting of county hospitals, township hospitals, and village clinics, guarantees country-dwellers essential health services. As the pinnacle of the service network, county hospitals represent the local top level of medical care delivery. It could be argued that the efficiency of county hospital medical services is directly related to the medical and health security of hundreds of millions of Chinese rural residents (1–3). However, in terms of service capacity and efficiency, county-level hospitals are still far from urban tertiary hospitals, and the development levels of county hospitals are also uneven (3–5). Improving the delivery efficiency of county-level hospitals can effectively improve the economy of regional health resource allocation. From a worldwide perspective, a large number of empirical studies show that the low efficiency of grass-roots medical institutions is a common situation, especially in a region with lower medical and health development, which leads to the unreasonable allocation of health services and the waste of medical resources (4–7). This dilemma has perplexed the central and local health administrative departments. In China, the central administration released the action plan for the Promotion of High-Quality Development (PHQD) of public hospitals in 2021, which proposes to enhance the comprehensive capabilities of county-level hospitals further and achieve PHQD (8). Service efficiency has become an essential issue in PHQD for public hospitals. Therefore, research on service efficiency's key factors can help provide decision-makers references for policymakers, thus providing the evidence-based basis for determining regional health policies.

Many scholars have studied the efficiency of health institutions in different countries, focusing on the measurement methods of hospital efficiency and its determinants (9). The Data Envelopment Analysis (DEA) and Stochastic Frontier Analysis (SFA) are widely used in terms of efficiency measurement (10–12). As a non-parametric method, DEA is particularly suitable for discussing the efficiency measurement of multi-input and multi-output scenarios (13–15). For example, the undesirable output model (16), bootstrapping approach (17–20), multi-stage analysis (7, 21, 22), and other DEA methods are often used in the literature on hospital efficiency research. In discussing the key factors of hospital efficiency, regression models were the prevailing integrated approach with DEA. Since the value calculated by the DEA was between 0 and 1 and most of the scores mainly concentrated on their boundary, most scholars used Tobit regression to explore the influence of variables on efficiency (23). However, the literature review shows that the research on the factors influencing hospital efficiency still has the following deficiencies.

Firstly, there is a lack of a systematic discussion framework on the variables affecting efficiency. It is not noticed that efficiency factors exist in the internal organizational structure, medical behavior, and the complex external environment (24). Secondly, most studies use the method of Tobit regression to discuss the contributing factors, ignoring the multiple causalities between these factors and efficiency results (23, 25). However, the actual situation is that various factors affect each other and influence operation efficiency. There were existing limitations in exploring the net effect of a single variable on outcomes. It is challenging to explain causality using correlation analysis. Finally, the study only tells readers which factors are significant and lacks specific suggestions for the efficiency improvement of different hospitals (10). The environment and organizational structure of various hospitals may be highly heterogeneous, and the efficiency improvement measures of each hospital may be other.

Therefore, based on the case-oriented Qualitative Comparative Analysis (QCA) method, this study explores the multiple configuration paths of the contributing factors to county-level hospitals' efficiency and provides evidence-based suggestions for efficiency progress. To the best of our knowledge, this is the first study to analyze the antecedent configuration of county-level hospital efficiency. Thus, this paper sheds new light on the discussion of influencing elements of hospital efficiency and efficiency improvement measures from a holistic perspective.

The rest of the study is organized as follows. Section Methods and Materials introduces the theoretical basis and analysis methods of this study and explains the samples, variables, and data sources. Section Results reports the efficiency measurement results of the case hospital and the critical steps in the qualitative comparative analysis, including efficiency value calibration, necessary condition analysis, standard analysis, and robustness test. Section Discussion discusses the configuration path of county-level hospital efficiency influencing factors. Section Conclusion summarizes these findings and clarifies the highlights and limitations of this study.

## Methods and Materials

### Bootstrap-DEA Model

The measurement of hospital efficiency is a classic topic in Health Economics. In terms of methodology, the current academia divided it into two categories, namely parametric and non-parametric methods (10). The non-parametric approach, represented by the DEA method, was widely used because of the multi-input and multi-output nature of the healthcare system (26), which originated from Farell's concept of technical efficiency and was proposed by Charnes et al. (27). So far, the method has been widely used in measuring the efficiency of healthcare delivery systems in both developed and developing countries (28–32). Since the DEA approach creates an efficiency frontier based on available data, the efficiency values calculated using this method are inherently biased in a positive and or at least non-negative direction (13). The bootstrapping and jackknife methods were often used as a means of repeated sampling to solve the problem of serially correlation estimates (14, 23, 26, 33). In this study, the output-orientated Constant Returns to Scale (CRS) DEA method was used to measure the sample hospitals' original technical efficiency because the demand for quality medical resources exceeds the supply at the county level in China. Subsequently, the bootstrapping technique (34) was used to correct the efficiency values, and those scores were defined as the outcome variable of the QCA method. The confidence interval method was based on the following formula: Percentiles of the original score/(1+ bootstrapped Bias/original score) (35). Two thousand times were selected to improve the accuracy of the value correction during bootstrapping process. MaxDEA Ultra (Version 7.9, Realworld Corp., Beijing, China) was employed to carry out the efficiency calculation.

### Qualitative Comparative Analysis Method

In this study, the fuzzy-set QCA (fs-QCA) method was used to analyze the configurational paths of efficiency advancement in a sample of hospitals. To the best of our knowledge, few studies applied this method to discuss the factors contributing to the efficiency of service delivery in healthcare sectors. As a new approach beyond qualitative and quantitative research, QCA offers a unique perspective in explaining the antecedent conditions that influence the emergence of a given outcome (36). Unlike regression models that examine the net effects of individual variables, this approach was based on the set theory of Boolean algebra. It enables a holistic perspective to explore complex social problems with multiple concurrent outcomes by examining the sufficient and necessary subset of relationships between antecedent conditions and outcomes (37). In dealing with complex causal issues, the QCA approach adheres to various possible paths to achieve the desired outcome, a feature often understood as all roads lead to Rome (38). QCA was divided into three categories depending on the set form: crisp sets, multi-valued sets, and fuzzy sets (36). Unlike the crisp sets' dichotomy, the fuzzy set allows partial membership scores with variable values between 0 and 1. The fuzzy set score represents the degree of a set of different cases, including two qualitative states, namely, full membership and full non-membership (38). Therefore, fuzzy-set QCA is entirely appropriate for discussing the causal relationship between hospital technical efficiency and its antecedent variables.

Moreover, since the method is based on set theory, it is particularly suitable for small and medium-sized samples selected on a case-oriented basis (38). When using this method for analysis, Ragin points out that QCA emphasizes the importance of identifying research questions and selecting appropriate conditions and outcome variables based on theoretical or empirical knowledge (38).

**Figure 1:**
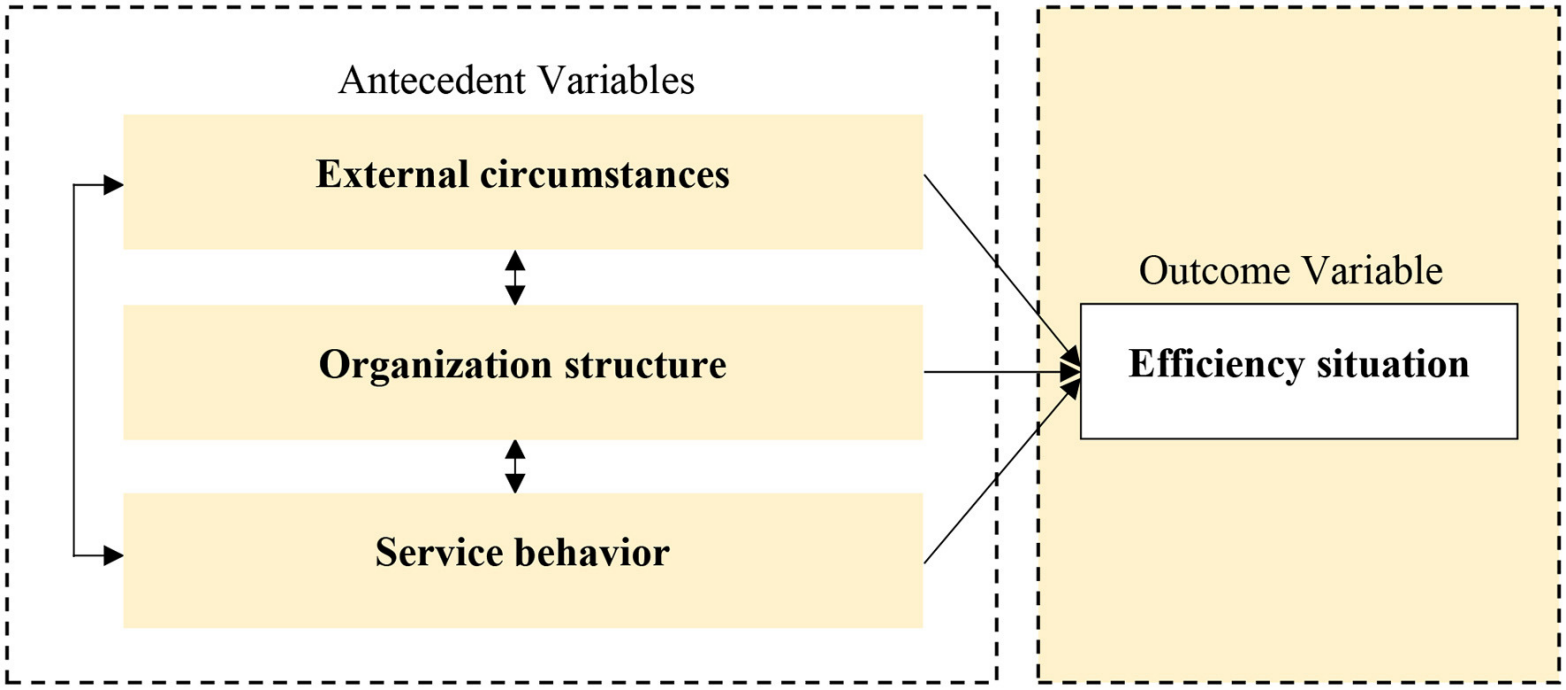
Environment-structure-behavior analytical framework.

Through the literature review, critical factors affecting hospital efficiency can be divided into three categories: external circumstance, organizational structure, and service behavior, as shown in Figure 1. Since the government and public institutions own public hospitals in China, the external environment, such as government subsidies, regional economic development level, and population size, become the key factors affecting the efficiency of public hospitals (5, 9, 39). In terms of hospital organizational structure, ownership type, hospital scale, and human resource structure were often used. In addition, as a healthcare service delivery unit, hospital service behavior also impacts its efficiency, including service capacity, bed utilization status, and patient structure (32, 40).

Therefore, an analytical framework based on Environment-Structure-Behavior (ESB) was constructed in this study and was used in the QCA approach to answering the multiple paths of efficiency states of county hospitals. Fs-QCA (Version 3.1b) software developed by Charles Ragin and Sean Davey was employed to carry out the QCA process.

### Variables and Data Source

In this study, 35 county hospitals in Hubei Province of China were selected as cases using a typical sampling method. Each hospital in the sample is the best medical institution in its county in terms of medical service capacity, and all of them meet the standards of Secondary Level A (SLA) general hospital and above as recognized by the National Health Commission, which meets the requirements for Decision Making Unit (DMU) homogeneity in the DEA method. In terms of variable selection, based on the literature review (13, 23), the number of doctors, registered nurses, actual open beds, and medical equipment (purchase price ≥ 1 million RMB) were selected as input indicators to reflect the input of human and physical resources in medical institutions. The number of outpatient and emergency visits, surgical operations for inpatient, and discharged patients adjusted by the Case Mix Index (CMI) and CMI were selected as output indicators to reflect the healthcare output of the medical institutions (26, 41). For setting antecedent variables, based on the ESB analysis framework, the annual per capita GDP of the county where the hospital is located and the yearly government financial subsidy were selected as proxies for the environmental dimension. The number of actual open beds and nurse-doctor ratio were used as proxy variables for the structural dimension. CMI and bed utilization rate were used as proxy variables for the behavioral dimension. The data in this study were obtained from the Hubei County Healthcare Comprehensive Reform Annual Report for the year 2019. The definitions of input indicators, output indicators, and antecedent indicators are illustrated in Table 1. The descriptive statistical analysis of the variables used in this study is shown in Table 2.

**Table 1:** Variables definition.

**Indicators**	**Variables**	**Definition**
Inputs	NoD	Number of doctors
	NoN	Number of nurses
	NoB	Number of actual open beds
	NoME	Number of medical equipment (Purchase price ≥ 1 million RMB)
Outputs	NoOEV	Number of outpatient and emergency visits
	NoSOI	Number of surgical operations for inpatient
	CMI	Case-mix index
	NoDP	Number of discharged patients adjusted by CMI
Antecedents	CCG	County per capita GDP (10 thousand RMB)
	AFA	Annual financial appropriation (1 thousand RMB)
	NoB	Number of actual open beds
	NDR	Nurse doctor ratio
	CMI	Case-mix index
	BUR	Bed utilization ratio

**Table 2:** Descriptive statistics of variables.

**Variables**	**Mean**	**Std. Dev**.	**Min**	**Max**
NoD	234	105	71	569
NoN	413	163	158	913
NoB	851	295	400	2,000
NoME	23	14	2	86
NoOEV	389,214	223,388	124,296	1,178,782
NoSOI	12,141	9,649	1,514	52,256
CMI	0.87	0.09	0.69	1.08
NoDP	33,023	14,796	12,347	89,180
CCG	5.26	3.21	2.15	17.68
AFA	23,677	25,164	1,810	112,431
NDR	1.84	0.30	0.98	2.66
BUR	100.71	11.73	81.66	134.53

## Results

### County Hospital Efficiency Scores

**Figure 2:**
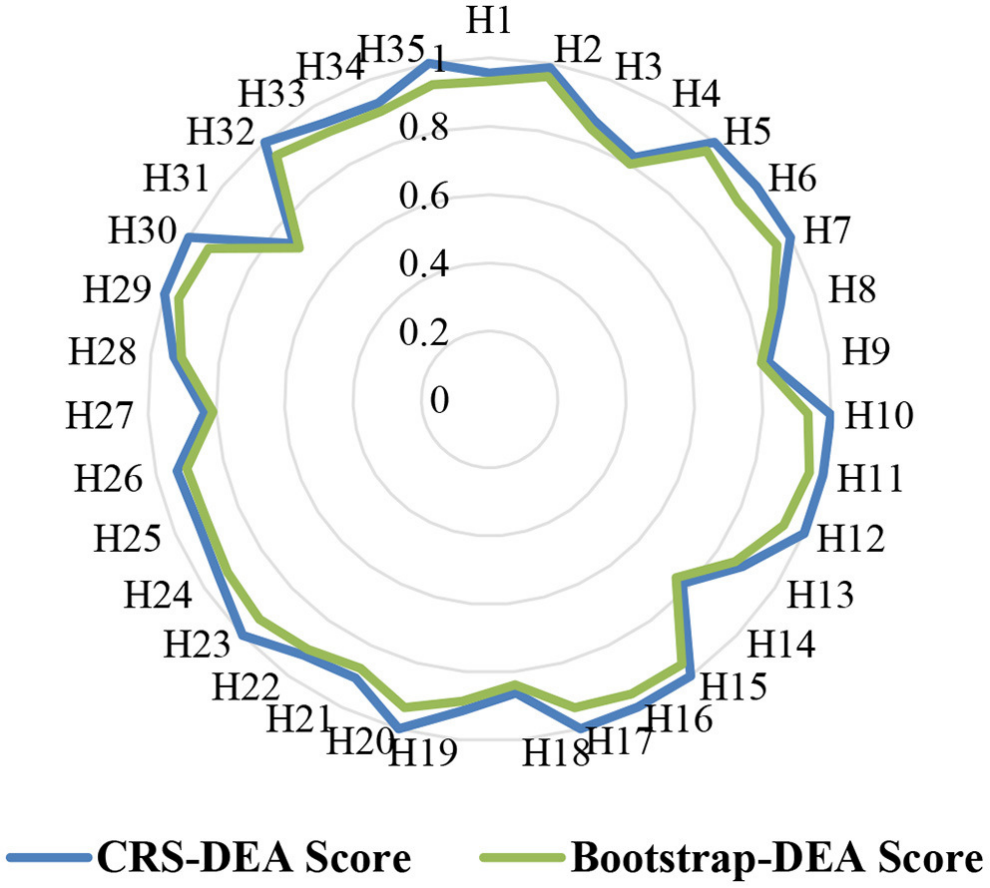
Technical efficiency score distribution radar chart.

The original efficiency scores of the 35 sample hospitals had a mean of 0.9376, a median of 0.9475, a standard deviation of 0.0729, a maximum value of 1, and a minimum value of 0.7316. After correction of bias, the efficiency values for hospitals showed a skewed distribution with a mean of 0.8994, a median of 0.9195, a standard deviation of 0.0621, a maximum value of 0.9662, and a minimum value of 0.7113. The efficiency scores of the sample hospitals all showed a decline after 2,000 replicate sampling. However, in terms of efficiency score average, they were still higher than the sample county hospitals in the studies by Li et al. (3), Cheng et al. (4), and Liu et al. (5). This difference may stem from the selection method of the sample hospitals. The technical efficiency score distribution of the sample hospitals before and after the correction of efficiency values is shown in Figure 2. Bootstrapping Efficiency Score (BES) was used as an outcome variable in the following QCA process.

### Variables Calibration for Fuzzy Sets

Fuzzy sets require converting metrics to sets and then calibrating them for full members, full non-members, and intersections (or maximum fuzzy points) in the set of interest (42). Qualitative anchor points determine the relationship between continuous variable scores and fuzzy set affiliation. Following Tang and Zhang (43, 44), each variable's 75th percentile, 50th percentile, and 25th percentile were considered fully membership, crossover point, and full non-membership. In this study, the antecedent and outcome variables of the case hospital were calibrated based on the calibration procedure in the fs-QCA, and the qualitative anchor points of the variables are shown in Table 3.

**Table 3:** Three qualitative anchors of each variable.

**Variables**	**Full membership**	**Crossover point**	**Full non-membership**
BES	0.936	0.920	0.872
CCG	6.532	4.300	3.040
AFA	30,114	14,567	7,829
NoB	1,000	800	692
NDR	2.012	1.848	1.664
CMI	0.92	0.86	0.80
BUR	105.188	98.387	93.774

### Necessary Conditions Analysis

The necessity of an outcome means that the condition must exist when the result occurs. The necessary conditions analysis of the outcome variable was first explored in the QCA. As we can see in Table 4, the consistency of all antecedent conditions was <0.9, which means that none of the antecedent conditions were necessary to achieve high or low levels of efficiency in county hospitals (42, 45, 46).

**Table 4:** Analysis of necessary conditions.

**Antecedent variables**	**High efficiency**		**Poor efficiency**	
	**Consistency**	**Coverage**	**Consistency**	**Coverage**
CCG	0.4515	0.5117	0.5659	0.5495
~CCG	0.6026	0.6183	0.4972	0.4371
AFA	0.4806	0.5279	0.5939	0.5589
~AFA	0.5984	0.6323	0.4983	0.4511
NoB	0.5009	0.5403	0.5426	0.5014
~NoB	0.5377	0.5784	0.5026	0.4631
NDR	0.5761	0.6313	0.4768	0.4477
~NDR	0.4960	0.5253	0.6073	0.5510
CMI	0.6446	0.6829	0.4111	0.3732
~CMI	0.4084	0.4473	0.6507	0.6107
BUR	0.5411	0.5975	0.5052	0.4780
~BUR	0.5272	0.5543	0.5746	0.5175

### Standard Analysis

Based on the calibration results, a standard analysis of the causal conditions arising from the results was performed. A data matrix (truth table) containing 2^k^ rows was constructed, where k is the number of causal conditions in the analysis. Each row of the matrix is associated with a specific combination of attributes and a high number of cases in this condition. At this stage, the minimum solution frequency was set to 1, and a more restrictive consistency threshold for the solutions was chosen to be 0.8 (42, 47). The consistency threshold was set beyond the 0.75 recommended by Ragin (38). Tables 5, 6 show the condition configurations to achieve high and poor efficiency in the county hospitals. The tables describe the relevant parameters for each configuration path, such as raw coverage, unique coverage, and overall solution coverage. According to Ragin and Fiss's explanation, the raw coverage is for the proportion of cases that satisfy this configuration, the unique coverage is the proportion of cases that uniquely satisfy this configuration but not any other configuration, and the overall solution coverage explains so the combined coverage of the configurations (38, 42). In addition, according to the parsimonious and intermediate solutions, the core and peripheral causal conditions are established in Tables 5, 6.

**Table 5:** Configuration of conditions for high efficiency.

**Configuration**	**Solution for high efficiency**				
	**C1**	**C2**	**C3**	**C4**	**C5**
CCG		⚫	⊗		⊗
AFA	⊗	⊗	⊗	⊗	•
NoB	⊗	⊗	•	•	⊗
NDR	•	•	⊗	•	⊗
CMI	⊗	⊗	•	•	⊗
BUR	⊗			•	•
Consistency	0.9211	0.8943	0.9414	0.9310	0.9131
Raw Coverage	0.1485	0.1257	0.1703	0.1438	0.0558
Unique Coverage	0.0414	0.0159	0.1083	0.0700	0.0287
Overall Solution Consistency	0.9219
Overall Solution Coverage	0.4203

*Full black circles and crossed-out circles indicate the presence and the absence of causal conditions, respectively. Large circles (⚫ and ⊗) indicate the core conditions, small circles (• and ⊗) indicate the peripheral conditions and the blank cells represent conditions that do not matter for the solution*.

**Table 6:** Configuration of conditions for poor efficiency.

**Configuration**	**Solution for poor efficiency**				
	**C1**	**C2**	**C3**	**C4**	**C5**
CCG		⊗	⚫	⊗	⚫
AFA	⊗	⚫	⚫	⊗	⚫
NoB	⊗	⚫	⚫	⚫	⊗
NDR	⊗		⊗	⊗	•
CMI	⊗	⊗	⊗	⊗	⊗
BUR	⊗	⊗		•	•
Consistency	0.8592	0.7840	0.8969	0.8032	0.9203
Raw Coverage	0.1096	0.1146	0.1239	0.1238	0.0786
Unique Coverage	0.0749	0.0676	0.0762	0.0675	0.0433
Overall Solution Consistency	0.8463
Overall Solution Coverage	0.4125

*The meaning of the symbols in this table has the same meaning as in Table 5*.

### Robustness Test

This study followed the method of Schneider and Wagemann (48) and White et al. (49) by changing consistency levels to test the robustness of the QCA results. When the consistency level was 0.85, the solution results were the same as in Table 5. when the consistency level was 0.72, the solution results were as shown in Table 7, with one additional configuration and no substantial changes in the main findings, except for minor changes in NoB and NDR, which indicated the relative robustness of the results of this study.

**Table 7:** Results of robustness test.

**Configuration**	**Solution for high efficiency**				
	**C1**	**C2**	**C3**	**C4**	**C5**	**C6**
CCG				•	⊗	⊗
AFA	⊗		⊗	⊗	⊗	⚫
NoB	•	•	⊗	⊗	⚫	⊗
NDR		•	⚫	⚫	⊗	⊗
CMI	⚫	⚫	⊗	⊗	⚫	⊗
BUR	⚫	⚫	⊗			⚫
Consistency	0.8401	0.8543	0.9211	0.8943	0.9414	0.9131
Raw Coverage	0.2648	0.2091	0.1485	0.1257	0.1703	0.0558
Unique Coverage	0.0456	0.0637	0.0414	0.0159	0.0414	0.0271
Overall Solution Consistency	0.8450
Overall Solution Coverage	0.5296

*The meaning of the symbols in this table have the same meaning as in Table 5*.

## Discussion

With the vigorous promotion of county healthcare reform in China, county-level hospitals have played a vital role in the system of tiered medical services with the treatment of major diseases without leaving the county and rehabilitation at the grassroots level. Although the classical regression method can identify the factors affecting the efficiency of county-level hospitals, it still faces difficulties in the specific practice of hospital management because of the complex internal and external circumstances.

This study provides a configuration perspective for understanding the multiple influences on county hospitals in China. The results of QCA indicate that the paths of high efficiency in county hospitals can be divided into three categories, five paths. We named it Structural Optimization (OP), Capability Enhancement (CE), and Government Support (GS), respectively. The consistency of each path is within the acceptable range (>0.8).

The OP type implies that the role of organizational structure optimization dominates this path (C1, C2, C4), illustrating the importance of optimizing the human resources structure in the efficiency improvement process without government support. As shown in Table 5, these three configurations are identical in terms of core conditions (NDR^*^~AFA) with second-order equivalence. The C1 condition suggests that regardless of the level of economic development of the county where the hospital is located, in the absence of government funding, the hospital can improve efficiency by increasing the NDR to optimize the human resource structure even in the presence of lower NoB, CMI, and BUR. However, if the hospital expands the NoB, it needs to improve CMI and BUR to obtain efficiency gains (C4). Meanwhile, when the hospital is in a county with a good level of economic development and lack of government support, regardless of BUR, the hospital can likewise improve efficiency by increasing the NDR when there is a low NoB and CMI (C2). It is important to note that a high NDR plays a central role in the efficiency improvement process in all three configurations. This was also confirmed in the study of Cheng et al. (4).

In China, inadequate NDR has been a dilemma for hospitals' development (50). In 2020, the nurse-physician ratio in rural areas in China was 1.02, lower than its city counterpart, 1.27 (51). Based on World Health Statistics 2020 released by WHO, the ratio of nurses (including midwifery personnel) to doctors in China was only 1.34, which falls more minor than that in the United States (5.57), Switzerland (4.08), Japan (5.04), and South Korea (3.09) (52). The number of nurses and nursing quality is directly related to public hospitals' operation efficiency (53). For example, when the number of nursing staff is insufficient and patients lack necessary care, the incidence of postoperative complications and mortality of patients after surgery will increase, increasing the hospital's adverse output and leading to low efficiency. Hence, the administrators should pay attention to the risk of nurse shortage in hospital management. In addition, nursing human resource policies and measures need to be enacted by the government health departments to enhance the nursing capacity of medical institutions.

The CE type indicates that in the context of a lack of economic environment and government support, hospitals need to improve their medical service capacity to obtain efficiency gains. The path of C3 shows that in the absence of a favorable external environment and with low NDR, upgrading the hospital's CMI can receive efficiency progress. Increasing the number of beds is an auxiliary condition. CMI is commonly used to measure the overall severity of diseases treated in hospitals and is an essential indicator of the level of hospital service capacity and one of the determinants of hospital efficiency, as demonstrated in the study using the truncated regression approach by Chowdhury and Zelenyuk (41). Currently, China is carrying out Diagnosis-related Group Prospective Payment System (DRG-PPS) reform within the context of public hospitals, so this pathway provides evidence-based recommendations for improving the efficiency of county-level hospitals under DRG payment. Meanwhile, from the functional orientation of county-level hospitals in the three-tier medical and health service system in rural China, the medical service capacity of county hospitals is also an inevitable requirement in its development to achieve that severe diseases without leaving the county in the goal of tiered medical services.

The GS type refers to the fact that when the level of economic development in the county is located is poor but government support is vital, increasing the bed utilization rate can effectively improve efficiency even if there exists lower NoB, NDR, and CMI (C5). The positive effect of bed utilization on technical efficiency was in line with a study in Canada and Iran (41, 54). The number of beds is an essential physical input resource for hospitals, and its usage can significantly affect the operational efficiency of hospitals. This reminds hospital managers that the utilization of bed resources is more important than the number of beds.

In this study, we also analyzed the grouping paths that generate poor efficiency in hospitals, intending to identify the antecedent conditions that lead to inefficiency and prevent hospitals from falling into it. As shown in Table 6, the consistency of all four configurations was acceptable, except for the consistency of C2 (0.7840), which did not meet the requirements of Fiss's study regarding consistency (42). Therefore, C2 was excluded from the solution for poor efficiency. By analyzing the four conditions, three categories, namely Insufficient Capacity (IC), Aggressive Expansion (AE), and Poor Decision-making (PD), were classified that leading to inefficiency in county hospitals.

The IC type indicates that low levels of NDR, CMI, and BUR play a core role in hospital inefficiency regardless of the level of economic development of the county in which they are located, while government support shortage and insufficient scale only play a supporting role (C1). This suggests that the hospital's poor human resource structure and misconduct in healthcare delivery play an essential role in the inefficiency type. This path revealed the pivotal role of poor structural and behavioral factors in the inefficient cluster of hospitals. Therefore, to avoid becoming a case in this configuration, structure and behavior indicators of hospital operation need sustained attention.

The AE type contains two configurations (C3, C4). This type elucidates the shortcomings of aggressive scale expansion exposed in service delivery. The path of the C3 shows that a large-scale increase in the number of beds can lead to inefficiency when the level of economic growth and government support in the county is good and when the NDR and CMI of the hospital are low. And C4 shows that a large beds scale increase also leads to inefficiency when the conditions of the level of economic development and government support in the county are lacking in the case of low NDR and CMI of hospitals. A high BUR assists this process. Consistent with the findings of Pirani, these two configurations present the disadvantages associated with the aggressive expansion of bed numbers in hospitals under different external environments (55). The medical cause of health in China is in a phase of rapid development, and the size of public hospitals is expanding at a high rate, as evidenced by a surge in the number of beds. Many studies have examined the appropriate size for the number of beds in Chinese public hospitals, confirming the inefficiencies that result from excessive scale (3, 4). The present type of configuration shows that under the premise of ignoring organizational construction, a rapid increase in the number of beds could lead to inefficiencies.

The PD type refers to the poor management strategy made by hospital managers under an excellent external environment that leads to hospital inefficiency. C5 shows that a low level of CMI became the core condition of inefficiency under the external environmental conditions with a high level of economic development and government support in the county. Meanwhile, high NDR and BUR became supporting conditions. This indicates that hospital managers lacked attention to medical service capability when facing a favorable external environment and did not realize that the level of medical service is the core competitiveness of the hospital. Therefore, neglecting CMI improvement is not advisable in hospital development, and managers need to note it.

## Conclusion

This study discusses the factors affecting county-level hospital efficiency from configuration. The results show that these factors influence and interact with each other, and the resulting configuration will lead to the efficient state of healthcare institutions. Therefore, the progress of hospital efficiency results from the joint action of multiple factors, not from the change of a single one. Three configuration types illustrated organizational structure factors with high-level NDR as the core condition that plays a crucial role in improving efficiency. Besides, the service capability factor with low-level CMI as the core condition has played a vital role in declining efficiency. Hence, each hospital needs to improve corresponding performance according to its situation and circumstance. Any measures aimed at efficiency promotion should be discussed within the framework of a case-specific analysis.

## Strengths and Limitations

Previous research has explored critical factors attributed to hospital efficiency of the net effect of independent variables on efficiency values. But we explored multiple causal relationships that lead to an efficiency state from holistic view. However, this study still has some limitations, such as the limited number of antecedent variables and lack of discussion of the efficiency antecedent configuration between natural years. Future research can start with overcoming the limitations of this study, for example, adopting panel data, including hospitals in different provinces, and discussing the effect of different combinations of antecedent variables on efficiency results in more detail. Besides, comparing the QCA results with the regression model would be an interesting direction.

## Data Availability Statement

The original contributions presented in the study are included in the article/supplementary material, further inquiries can be directed to the corresponding author/s.

## Author Contributions

GY was responsible for the study design, data analysis, and draft writing. GY, JN, YP, and JY were involved in manuscript revision. HT guided and supervised the research. All authors discussed the results, contributed to the article, and approved the submitted version of the manuscript.

## Funding

This research was supported by the National Natural Science Foundation of China (Grant Number: 71774061).

## Conflict of Interest

The authors declare that the research was conducted in the absence of any commercial or financial relationships that could be construed as a potential conflict of interest.

## Publisher's Note

All claims expressed in this article are solely those of the authors and do not necessarily represent those of their affiliated organizations, or those of the publisher, the editors and the reviewers. Any product that may be evaluated in this article, or claim that may be made by its manufacturer, is not guaranteed or endorsed by the publisher.
